# Excess non-COVID-19 mortality in Norway 2020–2022

**DOI:** 10.1186/s12889-023-17515-5

**Published:** 2024-01-22

**Authors:** Guttorm Raknes, Stephanie Jebsen Fagerås, Kari Anne Sveen, Pétur Benedikt Júlíusson, Marianne Sørlie Strøm

**Affiliations:** 1 Department of Health Registry Research and Development, Norwegian Institute of Public Health, Postboks 973 Sentrum, NO-5808 Bergen, Norway; 2 Raknes Research, Ulset, Norway; 3 Regional Medicines Information and Pharmacovigilance Centre (RELIS), University Hospital of North Norway, Tromsø, Norway; 4 Department of Health Registries, Norwegian Institute of Public Health, Bergen, Norway; 5 Department of Clinical Science, University of Bergen, Bergen, Norway; 6 Children and Youth Clinic, Haukeland University Hospital, Bergen, Norway

**Keywords:** COVID-19, Pandemic, Cause of death, Mortality, Register study, Norway, Norwegian cause of death registry

## Abstract

**Background:**

Causes of death other than COVID-19 seem to contribute significantly to the excess mortality observed during the 2020–2022 pandemic. In this study, we explore changes in non-COVID-19 causes of death in Norway during the COVID-19 pandemic from March 2020 to December 2022.

**Methods:**

We performed a population-based cross-sectional study on data from the Norwegian Cause of Death Registry. All recorded deaths from 1st January 2010 to 31st December 2022 were included. The main outcome measures were the number of deaths and age-standardised death rate (ASMR) per 100000 population from the major cause of death groups in 2020, 2021 and 2022. The predicted number of deaths and ASMRs were forecasted with a 95% prediction interval constructed from a general linear regression model based on the corresponding number of deaths and rates from the preceding ten prepandemic years (2010–2019). We also examined whether there were deviations from expected seasonality in the pandemic period based on prepandemic monthly data from 2010–2019. The cumulative number of deaths and ASMR were estimated based on monthly mortality data.

**Results:**

There was significant excess mortality (number of deaths) in 2021 and 2022 for all causes (3.7% and 14.5%), for cardiovascular diseases (14.3% and 22.0%), and for malignant tumours in 2022 (3.5%). In terms of ASMR, there was excess mortality in 2021 and 2022 for all causes (2.9% and 13.7%), and for cardiovascular diseases (16.0% and 25,8%). ASMR was higher than predicted in 2022 for malignant tumours (2.3%). There were fewer deaths than predicted from respiratory diseases (except COVID-19) in 2020 and 2021, and from dementia in 2021 and 2022. From March 2020 to December 2022, there were cumulatively 3754 (ASMR 83.8) more non-COVID-19 deaths than predicted, of which 3453 (ASMR: 79.6) were excess deaths from cardiovascular disease, 509 (ASMR 4.0) from malignant tumours. Mortality was lower than predicted for respiratory diseases (-1889 (ASMR: -44.3)), and dementia (-530 (ASMR -18.5)).

**Conclusions:**

There was considerable excess non-COVID-19 mortality in Norway from March 2020 until December 2022, mainly due to excess cardiovascular deaths. For respiratory diseases and dementia, mortality was lower than predicted.

**Supplementary Information:**

The online version contains supplementary material available at 10.1186/s12889-023-17515-5.

## Introduction

The COVID-19 pandemic resulted in the largest surge in deaths since World War II in Norway [1].

It is generally presumed that the SARS-CoV-2 virus caused most of the extra deaths, but it has been increasingly evident that not all the excess mortality can be ascribed to registered COVID-19 deaths [2, 3]. There have been concerns that restrictions may have had negative impact on other diseases, partly because patients have avoided seeking necessary medical attention [4]. Mental health issues due to isolation may have had wide-ranging consequences, even for somatic conditions. On the other hand, there is reason to believe that infection control measures may have had beneficial effects on the outcome of some conditions [5].

Public health authorities in European countries have generally provided reliable, relevant, and immediately updated statistics on the number of people infected and dying from COVID-19. Changes in mortality from other causes have not been subject to the same extensive scrutiny. We are far from yet to see the full picture of how the COVID-19 pandemic affected the mortality of other causes.

We wanted to examine how mortality from cardiovascular and respiratory diseases, dementia, cancer, and external causes developed in Norway during the pandemic years 2020–2022 compared to trends in the preceding years.

The objective of this study was to investigate whether there was a higher or lower mortality than expected for any of the main groups of causes of death in Norway during the pandemic until December 2022 based on cause of death register data.

## Methods

We conducted a cross-sectional, observational, population-based register study.

Data were obtained from the Norwegian Cause of Death Registry and included information from all death certificates between 2010 and 2022 received by the 11th of May 2023.

The study included all deaths occurring from 1st January 2020 until 31st December 2022 and all deaths from 1st January 2010 to 31st December 2019 as the reference. The data included Norwegian residents who died in Norway and abroad, but not foreign citizens who died in Norway.

The main outcomes of interest were the number of deaths and age standardised mortality rates (ASMRs, per 100000 inhabitants) for 2020, 2021, and 2022 for all causes and for the major groups of causes of death: malignant neoplasms (cancer) (C00-C97), cardiovascular diseases (I00-I99), dementia (F01, F03, G30), respiratory diseases (J00-J99), and external causes (V01-Y98). External causes are deaths not caused by disease and include accidents, intentional and nonintentional injuries, and poisonings. Respiratory disease does not include COVID-19. The number of deaths and ASMR were also calculated for deaths from COVID-19 (U07.1-U07.2, U10.9), deaths due to adverse effects of COVID-19 vaccines (U12.9), and from cases without a registered death certificate (cause of death unknown) and for all other remaining causes. Separate analyses for all non-COVID-19 causes of death (all causes except COVID-19) were also performed. Although both the number of deaths and ASMR often follow similar trends, we have chosen both as outcome measures: The number of deaths is important for measuring the absolute burden on the Norwegian population and the healthcare system, while ASMR is important for being able to compare mortality rates between countries with different age distributions.

Secondary outcomes include monthly number of deaths and ASMR for the same abovementioned cause of death groups, compared with predicted values with 95% prediction intervals based on extrapolation of each month for the prepandemic years 2010–2019. The deviation from the predicted number of deaths and ASMR for each month were added chronologically and cumulatively for the pandemic period starting in March 2020. The first COVID-19 death in Norway was registered on the 12th of March 2020, and the first lockdown started two days later.

Doctors report deaths to the Population Register and causes of deaths to the Norwegian Cause of Death Registry by completing a death certificate following WHO’s international standard. Until 2018, all deaths were reported using a paper form (1993 version) [6]. From September 2018, an online electronic version of the death certificate became available [7]. Paper-based forms were gradually phased out, and from the 1st of January 2022, the use of the electronic death certificate became mandatory.

The ICD-10 code of the underlying cause of death in the register is determined from the death certificate by an automated coding system (IRIS) based on the Automatic Classification of Medical Entry (ACME) software [8, 9]. When needed, the cause of death is manually evaluated by trained staff from the Norwegian Cause of Death Registry. The term “cause of death” used throughout this manuscript is equivalent to the “underlying cause of death” as defined by the WHO [10].

### Missing data and bias

Although mandatory by law, not all causes of death are reported to the Norwegian Cause of Death Registry. In a typical year, approximately two percent of deaths have unknown causes. To achieve as complete coverage as possible, reminders were routinely sent to the municipality medical officers when paper forms were not received within a reasonable time. For electronic death certificates, an automatic reminder was sent the reporting doctor if the cause of death was not received within 6, 10 and 60 days. The introduction of the electronic death certificate contributed to speeding up the logistics and to reducing the number of missing causes of death. The cause of death register receives electronic certificates within 24 h with no need for postal service or manual processing.

The electronic death certificate is only available to Norwegian doctors. Norwegian citizens residing in Norway who die abroad are historically overrepresented among deaths with unknown cause. Unfortunately, the National Population Register stopped recording the geographical location of deaths at the end of 2020, and after this, it has not been possible to perform separate analyses of deaths that occurred abroad.

Additionally, in cases where an autopsy is necessary to determine the cause of death, there are major delays in reporting. Data for one year are considered complete by publication of the official annual Norwegian cause of death statistics, typically six months into the next year, but data are continually updated if new information is received.

We made a comparison of various basic characteristics of deaths with and without a known cause of death before and during the pandemic to clarify whether this led to significant biases.

### Statistical methods

Handling of data and statistical analyses were carried out in Toad for Oracle and Microsoft Excel. The number of deaths and ASMRs for 2020, 2021, and 2022 were compared with data from 2010 to 2019. Age-standardised mortality rates were computed by the direct standardisation method, using 5-year age strata and the European Standard Population of 2013 as the standard population [11]. The ‘at risk’ population was defined as the Norwegian mean population according to Statistics Norway for each year [12].

The observed number of deaths and death rates of 2020, 2021 and 2022 were compared with projections based on yearly data 2010–2019. Projections were estimated with linear regression and reported as a 95% prediction interval. Rates outside this interval were considered statistically significant changes. This means that the predictions were based on one-, two- and three-year extrapolations of linear trends for 2020, 2021 and 2022 respectively. To validate, we retrospectively predicted ASMRs for all causes, cancers, dementia, external causes, and cardiovascular and respiratory diseases for 1980–2019 based one-, two- and three-year extrapolations on 10-year linear trends. For 2010–2019 predictions, we calculated mean absolute error per cent (MAE%) and root mean square error per cent (RMSE%), and proportion outside the 95 per cent prediction intervals.

The number of deaths in all groups was expected to be more than large enough for normal distribution to be assumed (normal approximation of Poisson distribution). A Durbin-Watson test was used to detect autocorrelation among the annual observations.

The missing cause of death rates were determined by comparing the number of cases a with known cause of death with the number of registered deaths in the National Population Register. It is mandatory to be listed in the population register, and comparison is possible due to a unique personal identification number assigned to all Norwegian citizens. We assumed that causes of death were missing at random. To assess whether missing or delayed death certificates introduced bias, we examined the distribution of age and sex of the deceased and the location of death (home or hospital, nursing home). A similar comparison was made for causes of death based on electronic vs. paper-based death certificates.

## Results

In total, 537 757 deaths were reported in 2010–2022, of which 128,218 were reported in 2020–2022. There were 4112 cases of COVID-19 as the underlying cause of death, corresponding to 3.2 percent of all deaths in 2020–2022. Adverse effects of the COVID-19 vaccine were the underlying cause of death in 24 cases, of which 19 were in 2021 and five were in 2022.

Overall, the cause of death was missing in 9763 cases (1.8%). More details on missing data are found in Supplement 1. In 2020–2022, the cause of death was unknown in 2545 cases (2.0%).

Patient characteristics for deaths are presented in Table 1.

**Table 1:** Characteristics of persons dying in Norway 2010–2022. *N* Number of deaths, *IQR* Inter quartile range, *SD* Standard deviation

	**Mean** **2010–2019**		**2020**		**2021**		**2022**	
All deaths, N (Crude mortality rate, per 100000))	40954	(797.0)	40558	(753.9)	41713	(771.3)	45947	(842.0)
Mean age (SD)	79.0	(15.2)	79.5	(14.8)	79.8	(14.5)	79.9	(14.4)
Median age (IQR)	82.9	(17.8)	82.5	(17.2)	82.6	(16.7)	82.6	(16.5)
Females, N (%)	21128	(51.6)	20410	(50.5)	21352	(50.9)	22806	(50.1)
Deaths at home, N (%)	5697	(13.9)	6020	(14.8)	6292	(15.1)	6521	(14.2)
Hospital deaths, N (%)	12961	(31.6)	10800	(26.6)	11414	(27.4)	12850	(28.0)
Electronic death certificates, N (%)	131	(0.3)	14870	(36.7)	32914	(78.9)	43843	(95.4)
Cause of death missing, N (%)	722	(1.8)	1159	(2.9)	615	(1.5)	771	(1.7)

Mortality and excess mortality are presented in Table 2 (number of deaths) and Table 3 (ASMR) for the pandemic years 2020, 2021 and 2022. No significant autocorrelation was observed. There was significant excess all-cause mortality in 2021 and 2022, but in 2020, both the number of deaths and ASMR were within the 95 percent prediction interval. COVID-19 as a share of excess mortality was higher in 2021 than in 2022 both in the number of deaths (58% vs 49%) and as ASMR (72% vs 52%). Apart from COVID-19, excess mortality was most pronounced for cardiovascular diseases in both 2021 and 2022. The number of deaths from cancer was significantly but marginally elevated in 2022, but ASMR was within the 95% prediction interval in all pandemic years. The number of deaths from the “all other causes” group was significantly higher than predicted in all pandemic years, and a significant excess ASMR for this group was observed in 2021 and 2022. There were fewer missing causes of death (N) than predicted in both 2021 and 2022.

**Table 2:** Number of deaths in Norway 2020, 2021 and 2022 by major cause-of-death groups, COVID-19, and missing cause of death. Predictions and 95% prediction intervals are based on trends for 2010–2019. Significant excess mortality in bold and significantly lower mortality than predicted in italics

	**Year**	**Observed**	**Predicted**	**95% Prediction interval**	**Excess mortality**	**%**
**Malignant tumours**	**2020**	10876	10888	(10714 to 11063)	-12	-0.1
	**2021**	10962	10895	(10712 to 11078)	67	0.6
	**2022**	11282	10902	(10710 to 11094)	**380**	3.5
**Cardiovascular diseases**	**2020**	9577	9339	(8738 to 9939)	238	2.6
	**2021**	10205	8925	(8295 to 9555)	**1280**	14.3
	**2022**	10381	8511	(7849 to 9173)	**1870**	22.0
**Dementia**	**2020**	4067	4266	(3969 to 4563)	-199	-4.7
	**2021**	4265	4462	(4151 to 4773)	-197	-4.4
	**2022**	4442	4658	(4332 to 4985)	-216	-4.6
**Respiratory diseases**	**2020**	3768	4572	(4031 to 5112)	*-804*	-17.6
	**2021**	3660	4638	(4071 to 5205)	*-978*	-21.1
	**2022**	4401	4704	(4108 to 5300)	-303	-6.4
**External causes**	**2020**	2638	2604	(2375 to 2832)	34	1.3
	**2021**	2657	2613	(2374 to 2853)	44	1.7
	**2022**	2747	2623	(2371 to 2875)	124	4.7
**COVID-19**	**2020**	402	0	(0 to 0)	**402**	∞
	**2021**	852	0	(0 to 0)	**852**	∞
	**2022**	2858	0	(0 to 0)	**2858**	∞
**All other causes**	**2020**	8071	7721	(7410 to 8032)	**350**	4.5
	**2021**	8497	7703	(7377 to 8029)	**794**	10.3
	**2022**	9065	7685	(7342 to 8028)	**1380**	18.0
**Missing cause of death**	**2020**	1159	961	(652 to 1271)	198	20.6
	**2021**	615	1005	(681 to 1329)	*-390*	-38.8
	**2022**	771	1048	(707 to 1389)	*-277*	-26.5
**All non-COVID-19 causes**	**2020**	40186	40351	(39414 to 41288)	-165	-0.4
	**2021**	40861	40241	(39259 to 41224)	620	1.5
	**2022**	43089	40132	(39099 to 41165)	**2957**	7.4
**All causes**	**2020**	40558	40351	(39414 to 41288)	207	0.5
	**2021**	41713	40241	(39259 to 41224)	**1472**	3.7
	**2022**	45947	40132	(39099 to 41165)	**5815**	14.5

**Table 3:** Age standardised mortality rates (per 100000) in Norway 2020, 2021 and 2022 by the most important cause-of-death groups, COVID-19, and missing cause of death. Predictions and 95% prediction intervals are based on trends for 2010–2019. Significant excess mortality in bold and significantly lower mortality than predicted in italics than predicted in italics

	**Year**	**Observed**	**Predicted**	**95% Prediction interval**	**Excess mortality**	**%**
**Malignant tumours**	**2020**	223.6	225.3	(221.7 to 229)	-1.7	-0.8
	**2021**	220.2	221.0	(217.2 to 224.9)	-0.9	-0.4
	**2022**	221.6	216.7	(212.7 to 220.8)	**4.9**	2.3
**Cardiovascular diseases**	**2020**	199.8	193.3	(180.5 to 206.1)	6.5	3.4
	**2021**	208.6	179.7	(166.3 to 193.2)	**28.8**	16.0
	**2022**	209.0	166.2	(152 to 180.3)	**42.8**	25.8
**Dementia**	**2020**	86.1	92.0	(85.6 to 98.3)	-5.9	-6.4
	**2021**	88.7	95.4	(88.8 to 102.1)	*-6.7*	-7.1
	**2022**	90.9	98.9	(91.9 to 105.9)	*-8.0*	-8.1
**Respiratory diseases**	**2020**	78.8	97.0	(84.7 to 109.3)	*-18.2*	-18.7
	**2021**	75.0	96.9	(84 to 109.8)	*-21.9*	-22.6
	**2022**	88.1	96.8	(83.3 to 110.4)	-8.8	-9.1
**External causes**	**2020**	52.6	52.1	(47.2 to 57)	0.5	1.0
	**2021**	52.4	51.7	(46.5 to 56.8)	0.7	1.4
	**2022**	53.6	51.2	(45.8 to 56.6)	2.4	4.6
**Covid-19**	**2020**	8.7	0.0	(0 to 0)	**8.7**	∞
	**2021**	17.2	0.0	(0 to 0)	**17.2**	∞
	**2022**	57.5	0.0	(0 to 0)	**57.5**	∞
**All other causes**	**2020**	167.0	161.1	(153.8 to 168.4)	5.9	3.7
	**2021**	172.7	158.1	(150.4 to 165.7)	**14.6**	9.2
	**2022**	181.1	155.0	(147 to 163.1)	**26.0**	16.8
**Missing cause of death**	**2020**	23.8	19.7	(13.2 to 26.3)	4.1	20.6
	**2021**	12.2	20.4	(13.6 to 27.3)	*-8.3*	-40.5
	**2022**	14.9	21.1	(13.9 to 28.3)	-6.3	-29.6
**All non-COVID-19 causes**	**2020**	831.8	840.6	(819.1 to 862.1)	-8.8	-1.0
	**2021**	829.7	823.3	(800.8 to 845.9)	6.4	0.8
	**2022**	859.1	806.1	(782.4 to 829.7)	**53.0**	6.6
**All causes**	**2020**	840.5	840.6	(819.1 to 862.1)	-0.1	0.0
	**2021**	846.9	823.3	(800.8 to 845.9)	**23.6**	2.9
	**2022**	916.6	806.1	(782.4 to 829.7)	**110.6**	13.7

### Cumulative excess mortality

**Fig. 1:**
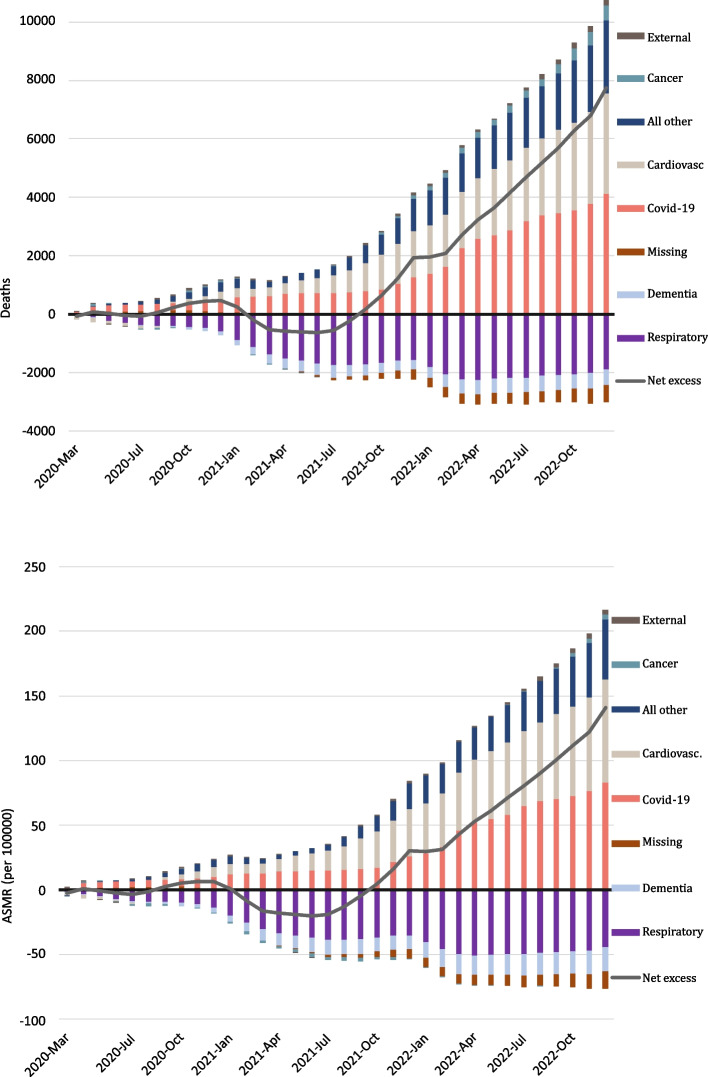
Cumulative excess number of deaths (top) and cumulative excess age standardised mortality rate (ASMR, bottom) by major cause of death groups. Norway April 2020 to December 2022. Bars below zero indicate a lower number of deaths/ASMR than predicted. The line indicates cumulative all-cause excess mortality

Monthly cumulative excess mortality in each group and combined from March 2020 (the first lockdown started on the 14th of March 2020) until December 2022 is presented in Fig. 1. There were fewer than predicted deaths until October 2021, after which there was a steadily increasing excess net mortality. Excess cumulative mortality was at its highest in December 2022 with 6684 (ASMR 83.0/100000). The lowest cumulative number of excess deaths was in June 2021, with -965 deaths, for ASMR the lowest was in July 2021 (-106.6 per 100000).

**Fig. 2:**
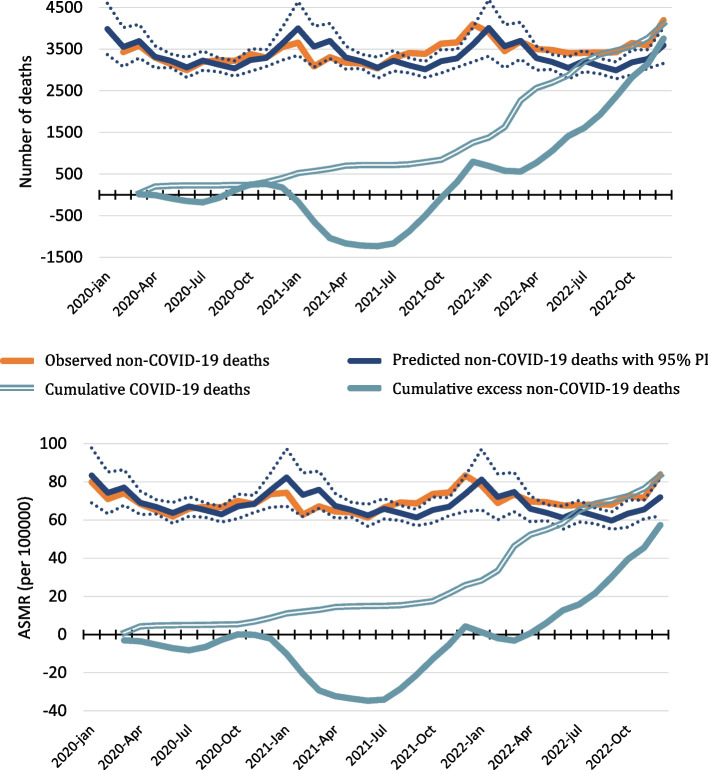
Monthly all-cause mortality in Norway 2020–2022. Observed and predicted with 95% prediction intervals (PI). Cumulative COVID-19 and excess non-COVID-19 deaths in Norway from March 2020 until December 2022 when the first lockdown was introduced. Number of deaths (top) and age-standardised mortality rates (ASMR) per 100000 (bottom)

**Fig. 3:**
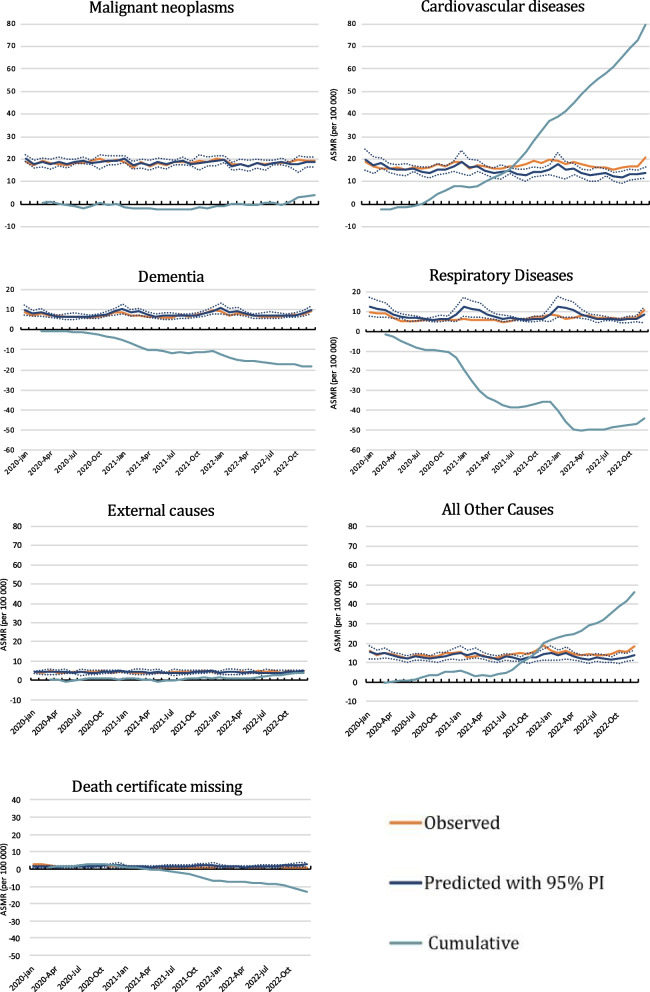
Monthly age-standardised mortality rates (ASMR) from malignant tumours (cancer), cardiovascular diseases, dementia, and respiratory diseases. Observed and predicted rates with 95% prediction intervals (PI). Cumulative excess ASMR in Norway from March 2020 when the first lockdown was introduced until December 2022

Monthly all-cause mortality was higher than predicted for all but three months between June 2021 and December 2022 (Fig. 2). Cumulative excess non-COVID mortality reached its lowest in June 2021, with 1356 fewer deaths than predicted (ASMR: -35/100000) (Fig. 3). For the remaining months until December 2022, the increase in the number of non-COVID deaths was 47% higher than the increase in cumulative COVID-19 deaths (ASMR: 35% higher).

By December 2022, non-COVID-19 causes comprised 47% of excess all-cause deaths (ASMR 41%) (Table 4). The excess cardiovascular ASMR was only 4.5% lower than the cumulative COVID-19 ASMR. There were 2419 fewer deaths than predicted from dementia and respiratory infections by December 2022, the same size as three-fourths of the cumulative COVID-19 ASMR.

**Table 4:** Cumulative excess mortality in major cause of death groups in Norway from March 2020 until December 2022. Number of deaths (N) and age standardised mortality rates (ASMR)

		**Cumulative excess mortality** **Mar 2020- Dec 2022**	**% of net excess mortality**
**Malignant tumours**	**N**	509	6.6
	**ASMR**	4.0	2.8
**Cardiovascular diseases**	**N**	3453	44.6
	**ASMR**	79.6	56.6
**Dementia**	**N**	-530	-6.8
	**ASMR**	-18.5	-13.1
**Respiratory diseases**	**N**	-1889	-24.4
	**ASMR**	-44.3	-31.5
**External causes**	**N**	199	2.6
	**ASMR**	3.6	2.6
**COVID-19**	**N**	4112	53.1
	**ASMR**	83.4	59.3
**All other causes**	**N**	2492	32.2
	**ASMR**	46.1	32.8
**Missing cause of death**	**N**	-602	-7.8
	**ASMR**	-13.2	-9.4
**All non-COVID-19 causes**	**N**	3754	46.9
	**ASMR**	83.8	40.7
**All causes**	**N**	7744	100.0
	**ASMR**	140.7	100.0

**Fig. 4:**
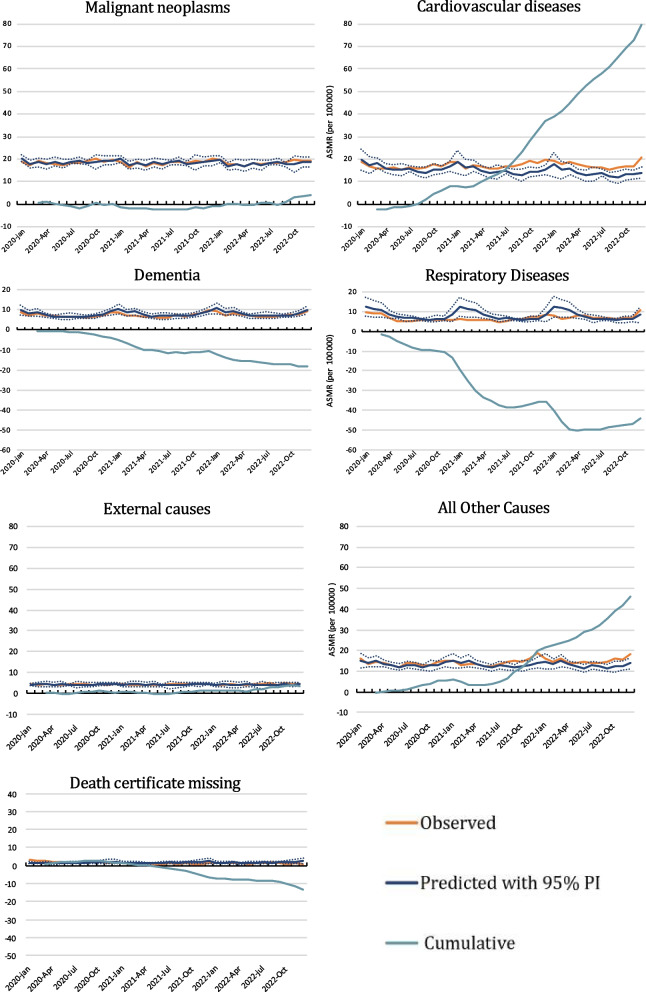
Monthly number of deaths from malignant tumours (cancer), cardiovascular diseases, dementia, and respiratory diseases, external causes, all other cases, and deaths without known cause (death certificate missing). Observed and predicted numbers with 95% prediction intervals. Cumulative excess number of deaths in Norway from March 2020 when the first lockdown was introduced until December 2022 when the first lockdown was introduced

As seen in Fig. 3 (ASMR) and Fig. 4 (N), cardiovascular causes dominated the excess non-COVID-19 deaths. There was a continuously higher and almost linearly increasing cardiovascular cumulative excess mortality between August 2020 and December 2022. The usual winter season increase in respiratory deaths was almost eliminated in 2020 and 2021, which corresponds to less than predicted mortality from these diseases. There was a similar but less pronounced trend for a lower than predicted dementia mortality.

Mortality from external causes was higher than predicted, although not significantly, in 21 of 34 months between March 2020 and December 2022. As seen in Table 4, this resulted in a small absolute excess cumulative mortality. There was net excess mortality for all other causes for almost all pandemic months until December 2022. There was also a significant reduction in deaths without known cause in 2021 and 2022.

The age and sex distribution among deaths with unknown cause of death was similar before and during the pandemic, but there were fewer deaths both at home and in hospitals (Table 5). There was a large drop in deaths without known cause registered as occurring abroad in 2020–2022 compared to the ten preceding years.

**Table 5:** Characteristics of deaths with or without death certificates (known or unknown cause of death). *N* Number of deaths, *IQR* Inter quartile range, *SD* Standard deviation

	**2010–2019**				**2020–2022**			
	**Known**		**Unknown**		**Known**		**Unknown**	
**N (%)**	402321	(98.2)	7218	(1.8)	125673	(98.0)	2545	(2.0)
**Females, N (%)**	208578	(51.8)	2706	(37.5)	63879	(50.8)	961	(37.8)
**Mean age (SD)**	79,2	(15.1)	69.9	(18.0)	79.9	(14.5)	72.9	(17.0)
**Median age (IQR)**	83.0	(17.6)	72.1	(23.9)	82.7	(16.7)	75.2	(20.6)
**Deaths abroad, N (%)**	1292	(0.3)	3901	(54.0)	101	(0.1)	248	(9.7)
**Deaths at home, N (%)**	56673	(14.1)	301	(4.2)	18833	(15.0)	0	(0.0)
**Hospital deaths, N (%)**	128928	(32.0)	679	(9.4)	34947	(27.8)	117	(4.6)

The validation of the predictions is presented in Supplement 2. For 2010–2019, MAE ranged from 1.2% to 6.5%, and RMSE was between 1.4% and 7.7%. The deviations were generally largest for the two-year extrapolations, and the predictions for respiratory ASMR had the highest MAE% and RMSE%. Out of the 180 predictions used for MAE and RMSE calculations, eight (4.4%) of observed ASMR values fell outside the corresponding 95% prediction interval.

## Discussion

### Key results

Excess non-COVID-19 mortality was seen in Norway from October 2021 through 2022 and cumulatively comprised 3632 (46.9%) of 7744 net excess deaths (ASMR 57.3, 40.7%). This was mainly driven by an increase in cardiovascular deaths, accounting for 44.6% of net cumulative excess deaths (ASMR 56.6%). A lack of seasonal increases during winter was observed for dementia and respiratory diseases in 2020 and 2021, which resulted in lower than predicted net cumulative mortality for dementia (N -6.8%; ASMR -13.1%) and respiratory diseases (N -24.4%, ASMR -31.5%). The number of cancer deaths was significantly but marginally higher than predicted in 2022 (3.5% of net excess deaths, ASMR 2.3%). Of the total net cumulative excess mortality, COVID-19 comprised the largest cause (N 51.1%, ASMR 59.3%). The remaining causes, including external causes, comprised 32.2% of net excess deaths (ASMR 32.8%) from March 2020 until December 2022.

### Limitations

Central Nordic health registers are generally considered to have a high coverage and contain reliable information. In the years observed in this study, the number of deaths in The Norwegian Cause of Death Registry without known cause of death varied between 577 and 1159, most of these residents dying abroad and where no certification on cause of death was sent to the registry. The number of unknown causes of death declined in 2021 and 2022, probably due to fewer Norwegians staying abroad and, more importantly, due to the introduction of electronic death certificates. From March 2020 to December 2022, there were 602 fewer deaths with unknown cause than predicted. As shown in Table 5, there is no indication of bias among these cases, except from country of death. We believe that if all causes of death were known, the distribution of causes of the excess mortality presented here would be only marginally different.

The use of electronic death certificates increased throughout the pandemic period. Total all-cause recorded mortality would not be affected by this, but the doctors filling in the certificates may tend to choose different causes of death when applying the electronic rather than the paper form. The reason is that the doctor could be nudged to choose certain diagnoses due to a predefined dropdown menu and a more standardised layout in the web-based solution. On paper, the causes of death were written without predefined options. We cannot rule out that the introduction of the electronic death certificate may have had an impact on the recorded incidence of certain causes of death, particularly in 2021 and 2022.

We cannot rule out that some causes of death were over- or underrepresented in death certificates received by the register after we extracted data for this study. We do not believe that the biases were sufficient to affect our main findings.

The information on COVID-19 status in the register is limited to cases where it was reported as an underlying or contributing cause of death. For example, we did not have any information about previous COVID-19 infection or vaccination status.

The pandemic itself may have influenced how doctors determined causes of death on the death certificate. For example, COVID-19 may have changed the level of precision for respiratory causes of death.

One could argue that predictions relying on two- and three-year extrapolations are questionable. Nevertheless, the validation results presented in Supplement 2 suggest that our method provides reliable predictions, even when extending the extrapolation to three years.Mortality displacement is a general challenge when analysing and interpreting excess mortality. From 2010 to 2019, there were no major deviations from mortality trends in Norway that should influence mortality in Norway significantly in the following COVID-19 pandemic years.

### Interpretation

The excess all-cause mortality in Norway found here is of the same magnitude but slightly higher than previously reported by the Norwegian Institute of Public Health [13]. This is also reflected in the fact that the reduction in life expectancy in Norway in 2022 was the largest since World War II [1]. Excess cardiovascular mortality during the COVID-19 pandemic has been observed in the USA [14] and Mexico [15]. In neighbouring Sweden and Denmark, there was no increase in cardiovascular mortality in 2020–2022 [16, 17]. 2020 and 2021 deaths from non-COVID-19 respiratory diseases were lower than predicted in Sweden.

It is possible that a substantial number of registered non-COVID-19 deaths were caused by undetected SARS-CoV-2 infections. There was an increase in excess non-COVID-19 deaths as well as COVID-19 deaths after mid-2021. The increased all-cause mortality coincides with the laxing of anti-infection measures, including lockdown, and with a decrease in routine COVID-19 testing. An increase in the number of deaths with unexplained causes in the USA has been observed both during influenza seasons and during the COVID-19 pandemic [18]. Some excess mortality with cardiovascular diseases and cancer, as well as “all other causes” as registered underlying causes of death, could probably be attributed to unintentional ignorance of COVID-19 infection by health personnel. It is also likely that the lifting of restrictions in 2021 led to an abnormally large spread of many respiratory infections in a population particularly susceptible after a long period without exposure to infectious agents.

Many people dying from non-COVID-19 causes at the end of the observation period in this study probably had a SARS-CoV-2 infection many months prior to, and apparently unrelated to their death. COVID-19 is a new disease, and thus far, little is known about what long-term effects the infection has on the course of other chronic diseases. The virus may have effects directly on different pathophysiological mechanisms that could lead to increased mortality. It is also likely that indirect effects on general mortality, such as peri- and postinfectious increased general frailty, malnutrition and wasting or long-COVID syndrome, may have contributed to increased mortality from several causes, including cancer.

There has been concern that lockdowns have resulted in less use of health care, leading to diseases that otherwise would have been discovered remaining undiagnosed, possibly with increased mortality. This may be one explanation for the accelerated reduction in several cardiovascular diseases in 2020 and 2021 according to the Norwegian Cardiovascular Disease Registry [19]. Routine follow-up consultations for chronic diseases may have been postponed or cancelled due to restrictions, or patients may have been reluctant to attend them due to fear of infection. Diabetes care may have suffered particularly, as increases in diabetes mortality have been observed in both Norway [20] and elsewhere [21]. Studies should be carried out to clarify whether there is an association between the underutilisation of health services and excess mortality from non-COVID-19 causes. Researchers should also investigate whether the restrictions have resulted in deterioration of lifestyle factors, such as less physical activity, a less healthy diet and even social and mental health issues that influence mortality.

There has been some opposition to mass vaccination during the COVID-19 pandemic due to concerns about potential harmful effects of allegedly insufficiently tested vaccines. There is a temporal concordance between increasing vaccine coverage and increasing excess mortality. From data available to us, it was not possible to compare excess mortality in vaccinated and unvaccinated individuals. Preliminary analyses from the National Preparedness Register for COVID-19 in Norway (Beredt C19), do not show any sign of increased mortality among vaccinated older people [22].

We have previously shown that compared to Sweden, Norway had a lower ASMR for cardiovascular and a higher ASMR for respiratory causes of death in 2010 to 2019 [23]. Some of these differences may not be fully explained by differences in morbidity between the countries. It is conceivable that there are different traditions in different countries for which causes of death doctors tend to indicate on the death certificate. The introduction of electronic death certificates may have led to Norwegian doctors changing the practice of filling in death certificates to more like how it is done in Sweden.

The lack of seasonal surges in respiratory infections in the winters of 2020/21 and 2021/22 probably contributed to some mortality displacement and thus more deaths than predicted for the rest of the observation period, but it cannot explain all of it.

Death often occurs in very old, multimorbid people, and it can be difficult to reliably determine the underlying cause of death. The lower than predicted dementia mortality may be partly due to fewer deaths from respiratory infections during lockdown.

### Generalisability

The generalizability of our findings to other countries should be considered in light of several factors. Although data from the Norwegian Cause of Death Register are known for high data quality and reliability, and thus has excellent internal validity, it is crucial to acknowledge that COVID-19 mortality rates in Norway were relatively lower compared to many similar countries. Furthermore, variations in testing intensity, vaccine coverage, and the stringency of public health measures make comparisons between countries less straightforward. In addition, there are differences in the age composition of the population in Norway compared to other nations. Therefore, while our results provide valuable insights into the Norwegian context, caution should be exercised when applying them to settings with distinct demographic profiles and pandemic response strategies.

## Conclusions

During the pandemic from 2020 to 2022, Norway experienced a significant increase in non-COVID-19 deaths. This rise in mortality, especially notable from late 2021 to December 2022, was primarily seen in cardiovascular diseases. To understand the underlying causes better, further research is essential to determine whether it resulted from the virus itself, the measures taken to control its spread, or other factors.

### Supplementary Information

**Additional file 1: Supplement 1.** The underlying data (monthly figures on the number of deaths and ASMR) can be found in the Excel workbook.

**Additional file 2: Supplement 2.** Validation of model. Retrospective predictions of age standardised mortality rates (ASMR) with 95% prediction intervals from 1980 to 2019. based on one-, two- or three-year extrapolations from preceding 10-year linear trends. Mean absolute error% (MAE%) and root mean square error% (RMSE%) for 2010-2019 ASMR predictions.

## Data Availability

The main analyses are based on cause of death data retrieved on the 11th of May 2023, and these data are found in Supplement 1. Updated data from the Norwegian Cause of Death Registry are publicly available online (http://statistikkbank.fhi.no/dar/, in Norwegian only). Monthly ASMR data are not available in the online database but are found in the Supplement 1. The online database is updated once a year, and late incoming death certificates provide gradually improving coverage. Data on the number of death certificates submitted electronically are not publicly available, but these will be made available on request. Weekly death statistics from the National Population Register can be accessed via Statistics Norway’s webpages (https://www.ssb.no/befolkning/artikler-og-publikasjoner/her-finner-du-ukentlige-tall-pa-antall-dode).

## Abbreviations

ASMR: Age-standardised mortality rate
Covid-19: Coronavirus disease 2019
IQR: Interquartile range
MAE: Mean absolute error
N: Number of deaths
PI: Prediction interval
RMSE: Root mean square error
SARS-CoV-2: Severe acute respiratory syndrome coronavirus 2
SD: Standard deviation
