# Supplementary material for: Excess non-COVID-19 mortality in Norway 2020–2022
Source: BMC Public Health. 2024 Jan 22;24:244. doi: 10.1186/s12889-023-17515-5 (PMC10801945; doi:10.1186/s12889-023-17515-5)

All causes

	Extrapolation		
	**1 year**	**2 years**	**3 years**
**Reference years**	-11 to -1	-12 to -2	-13 to-3
Outcome years in study	*2020*	*2021*	*2022*
Reference years in study	*2010-2019*	*2010-2019*	*2010-2019*
**Validation of predictions for 2010 to 2019**			
**MAE%**	1.2%	1.8%	1.4%
**RMSE%**	1.6%	2.2%	1.6%
**Outside 95% prediction interval**	0%	10%	0%

The table shows mean absolute error% (MAE%) and root mean square error% (RMSE%) for age standardised mortality rate (ASMR) predictions for the ten years preceding the COVID-19 pandemic, ie 2010-2019.

Predictions are based on extrapolation from linear 10-year trend until one-, two- or three years prior to each year predicted.

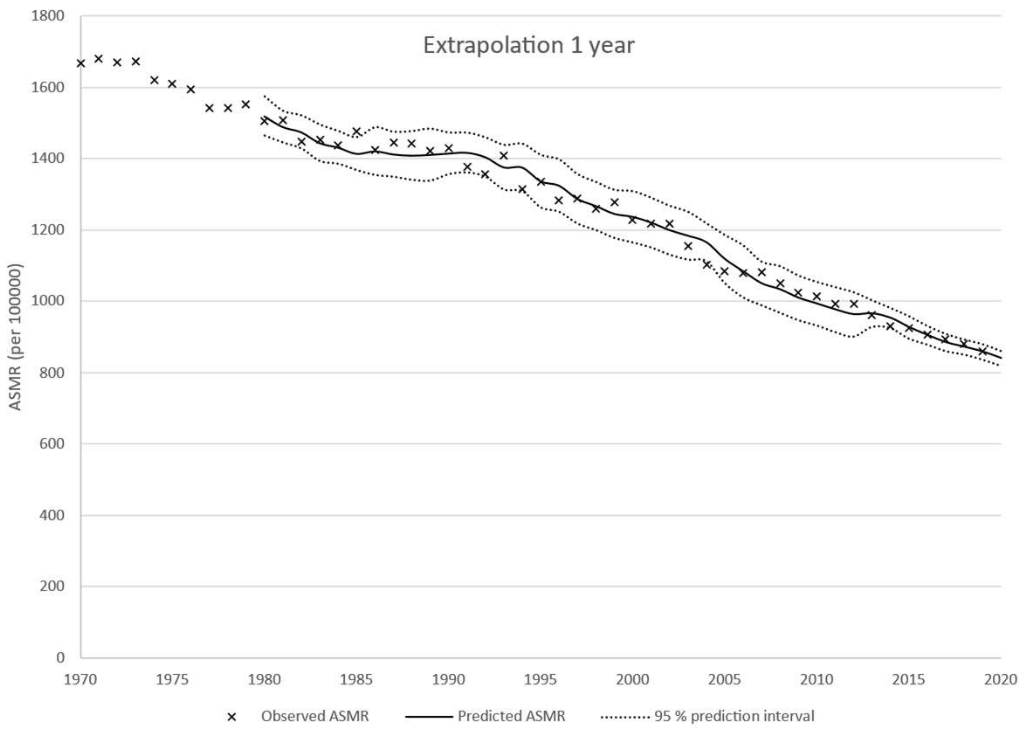

The figures display observed ASMR compared with moving predictions with 95% prediction intervals (PI) based on one-, two- or three-year extrapolations from preceding 10-year linear trends.

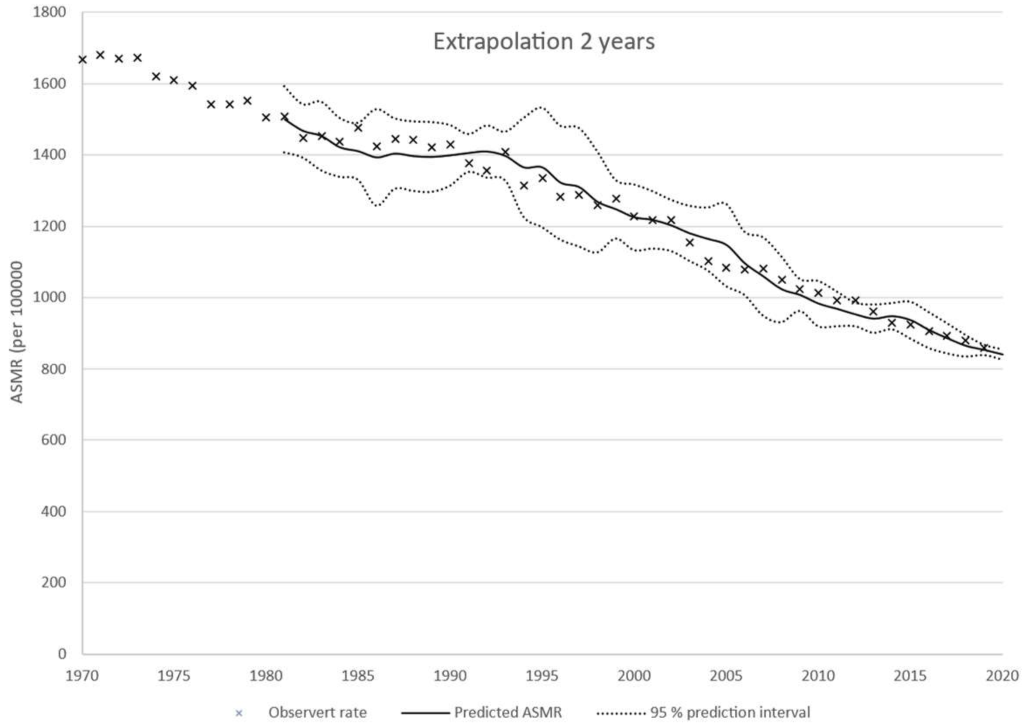

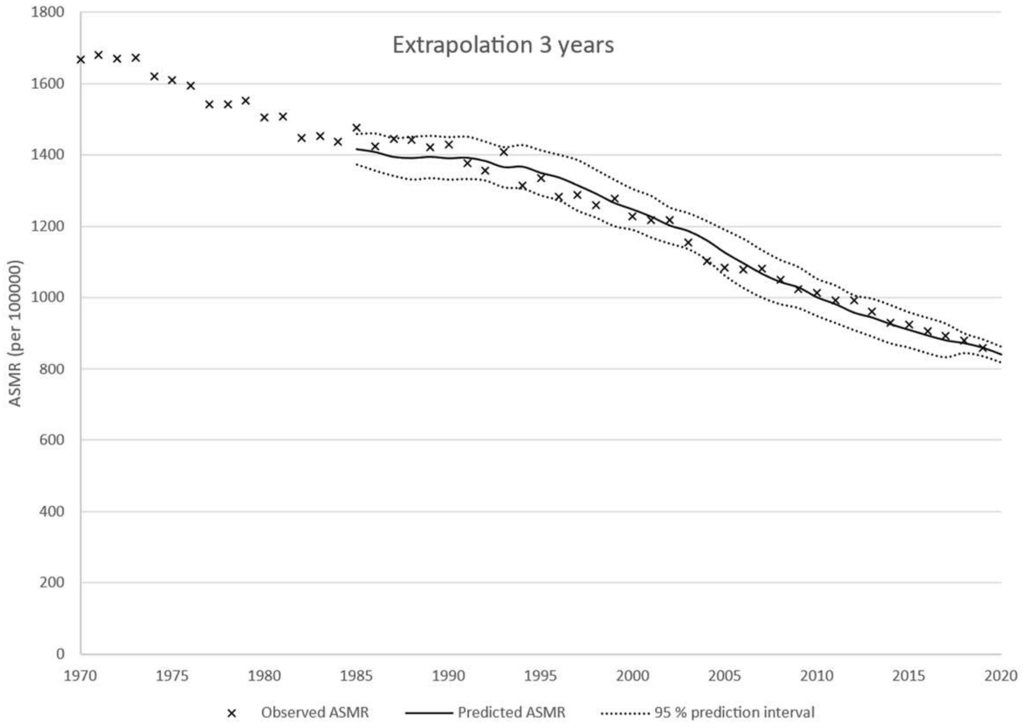

Malignant tumours (cancer)			
	**Extrapolation**		
	**1 year**	**2 years**	**3 years**
**Reference years**	-11 to -1	-12 to -2	-13 to-3
Outcome years in study	*2020*	*2021*	*2022*
Reference years in study	*2010-2019*	*2010-2019*	*2010-2019*
**Validation of predictions for 2010 to 2019**			
**MAE%**	1.2%	1.4%	1.5%
**RMSE%**	1.4%	1.6%	1.7%
**Outside 95% prediction interval**	0%	10%	0%

The table shows mean absolute error% (MAE%) and root mean square error% (RMSE%) for age standardised mortality rate (ASMR) predictions for the ten years preceding the COVID-19 pandemic, ie 2010-2019.

Predictions are based on extrapolation from linear 10-year trend until one-, two- or three years prior to each year predicted.

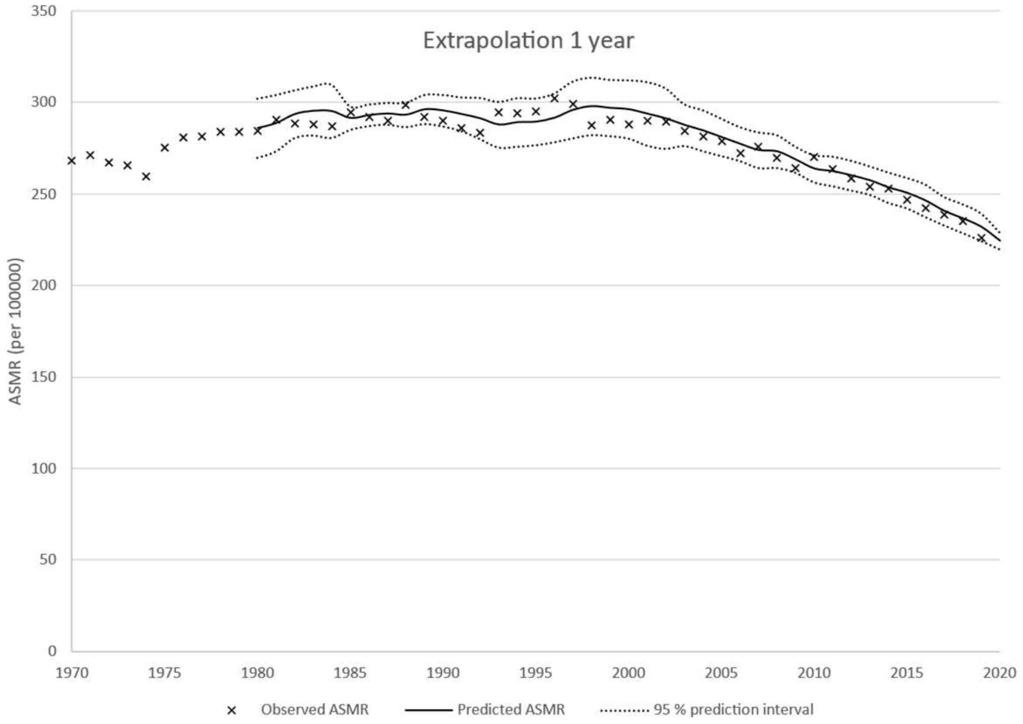

The figures display observed ASMR compared with moving predictions with 95% prediction intervals (PI) based on one-, two- or three-year extrapolations from preceding 10-year linear trends.

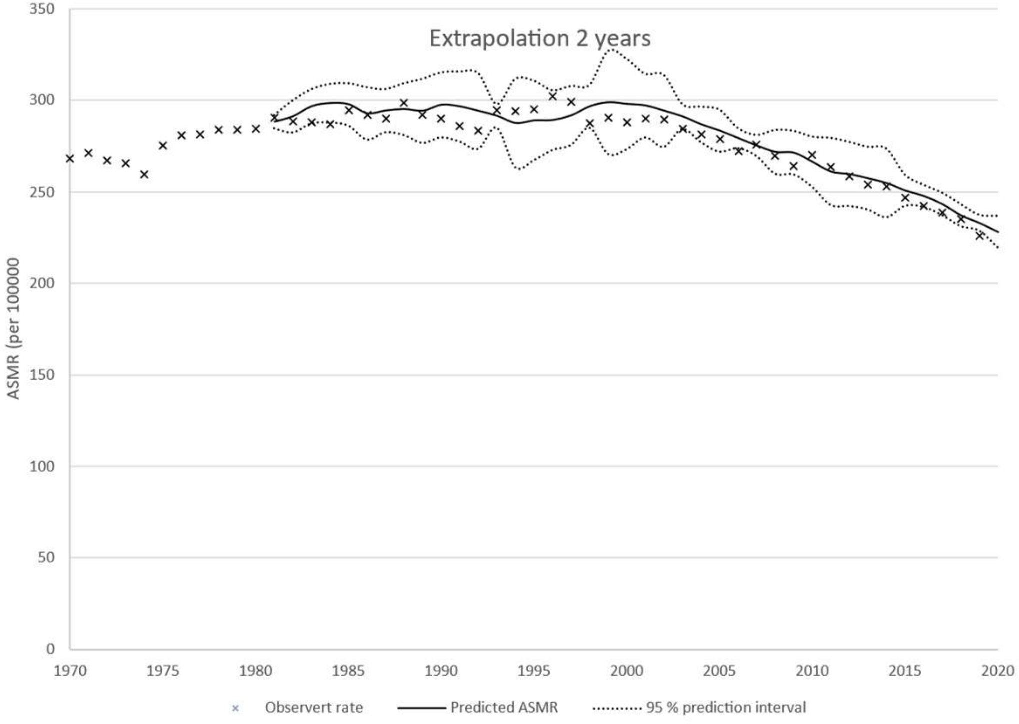

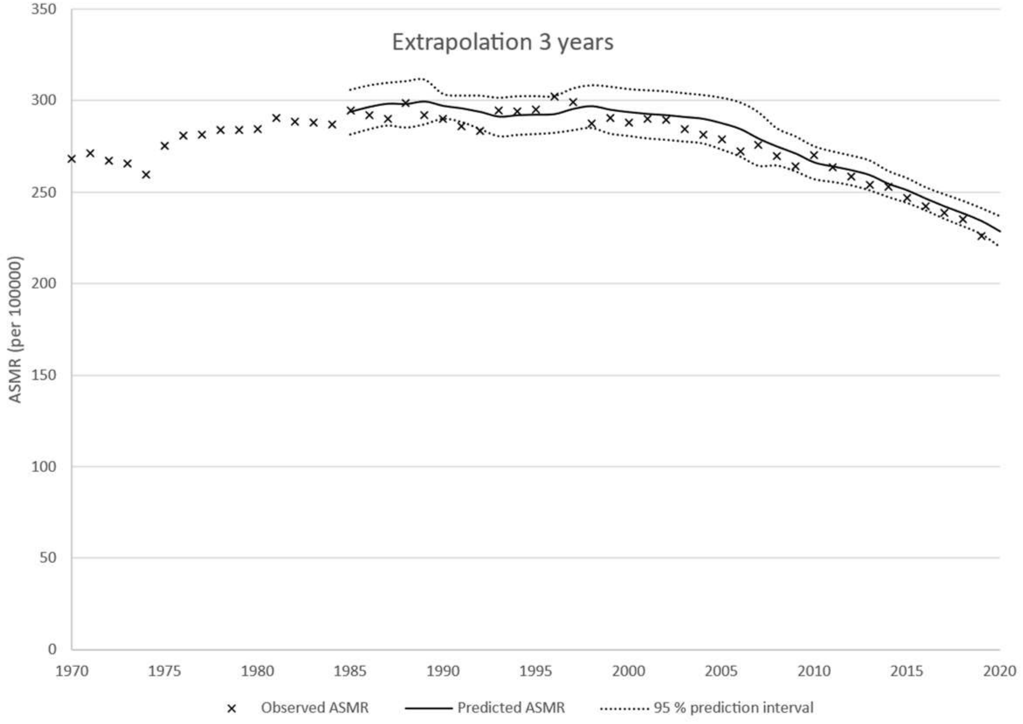

Cardiovascular diseases			
	Extrapolation		
	1 year	2 years	3 years
**Reference years**	-11 to -1	-12 to -2	-13 to-3
Outcome years in study	*2020*	*2021*	*2022*
Reference years in study	*2010-2019*	*2010-2019*	*2010-2019*
**Validation, predictions for 10 years (2010 to 2019)**			
**MAE%**	3.3%	5.3%	5.7%
**RMSE%**	4.7%	7.0%	6.8%
**Outside 95% prediction interval**	0%	20%	0%

The table shows mean absolute error% (MAE%) and root mean square error% (RMSE%) for age standardised mortality rate (ASMR) predictions for the ten years preceding the COVID-19 pandemic, ie 2010-2019.

Predictions are based on extrapolation from linear 10-year trend until one-, two- or three years prior to each year predicted.

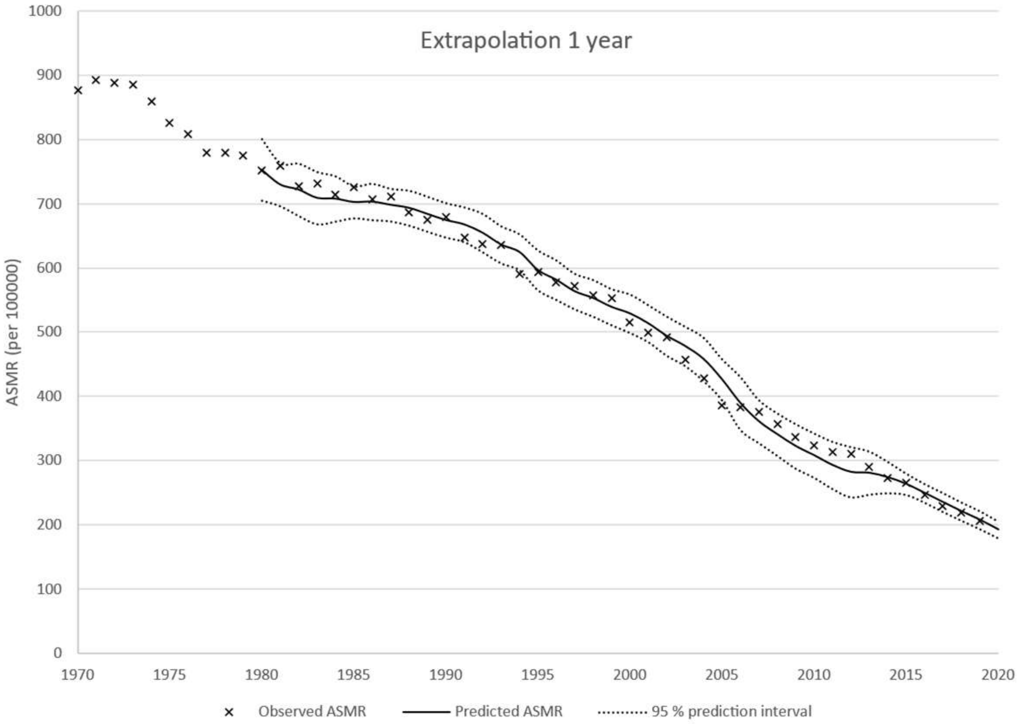

The figures display observed ASMR compared with moving predictions with 95% prediction intervals (PI) based on one-, two- or three-year extrapolations from preceding 10-year linear trends.

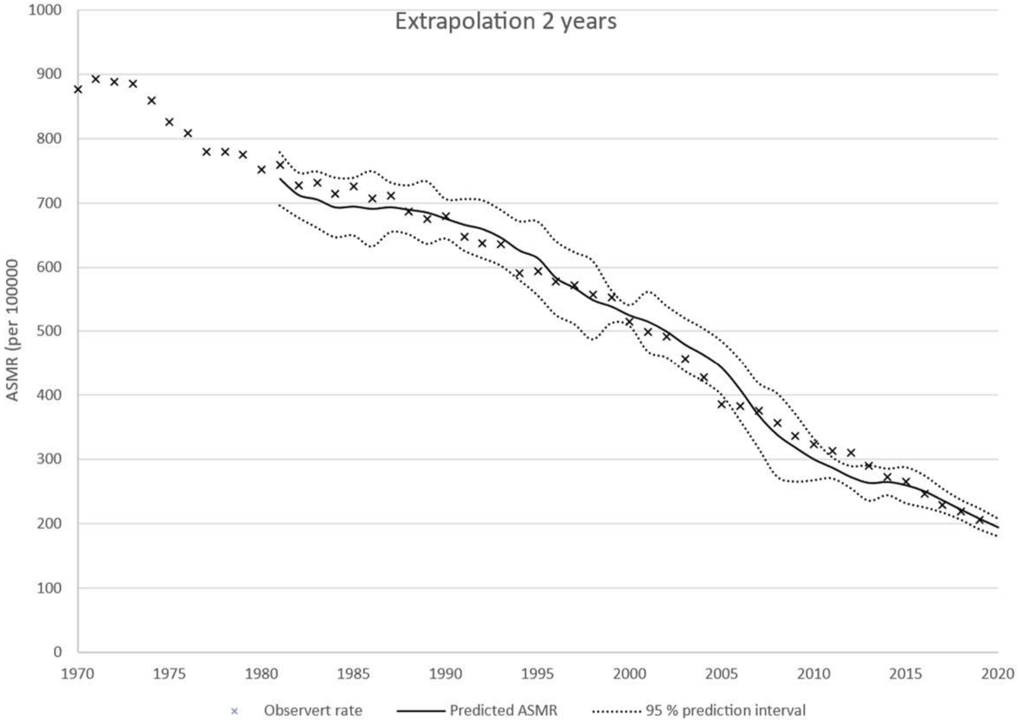

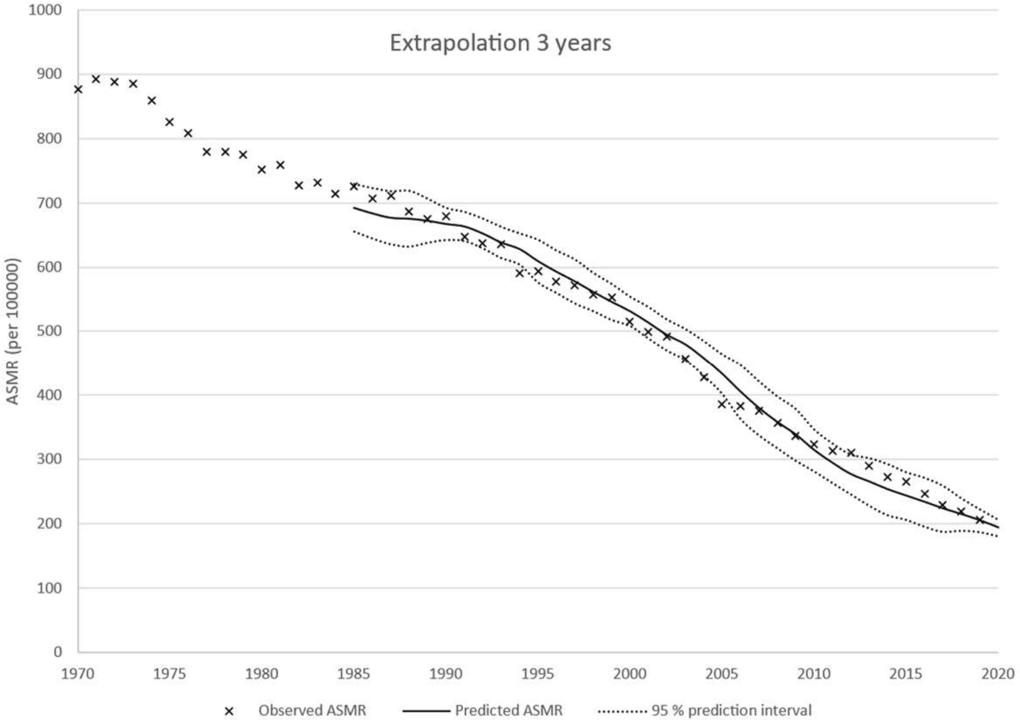

Respiratory diseases

	Extrapolation		
	**1 year**	**2 years**	**3 years**
**Reference years**	-11 to -1	-12 to -2	-13 to-3
Outcome years in study	*2020*	*2021*	*2022*
Reference years in study	*2010-2019*	*2010-2019*	*2010-2019*
**Validation of predictions for 2010 to 2019**			
**MAE%**	5.7%	6.5%	5.1%
**RMSE%**	6.6%	7.7%	6.4%
**Outside 95% prediction interval**	0%	10%	0%

The table shows mean absolute error% (MAE%) and root mean square error% (RMSE%) for age standardised mortality rate (ASMR) predictions for the ten years preceding the COVID-19 pandemic, ie 2010-2019.

Predictions are based on extrapolation from linear 10-year trend until one-, two- or three years prior to each year predicted.

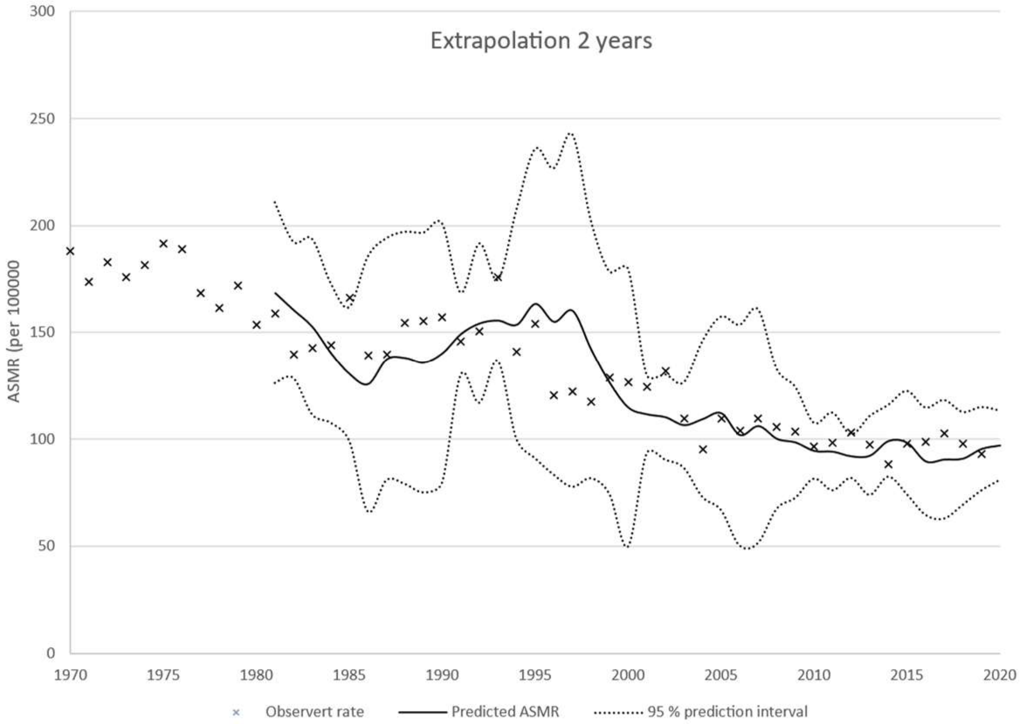

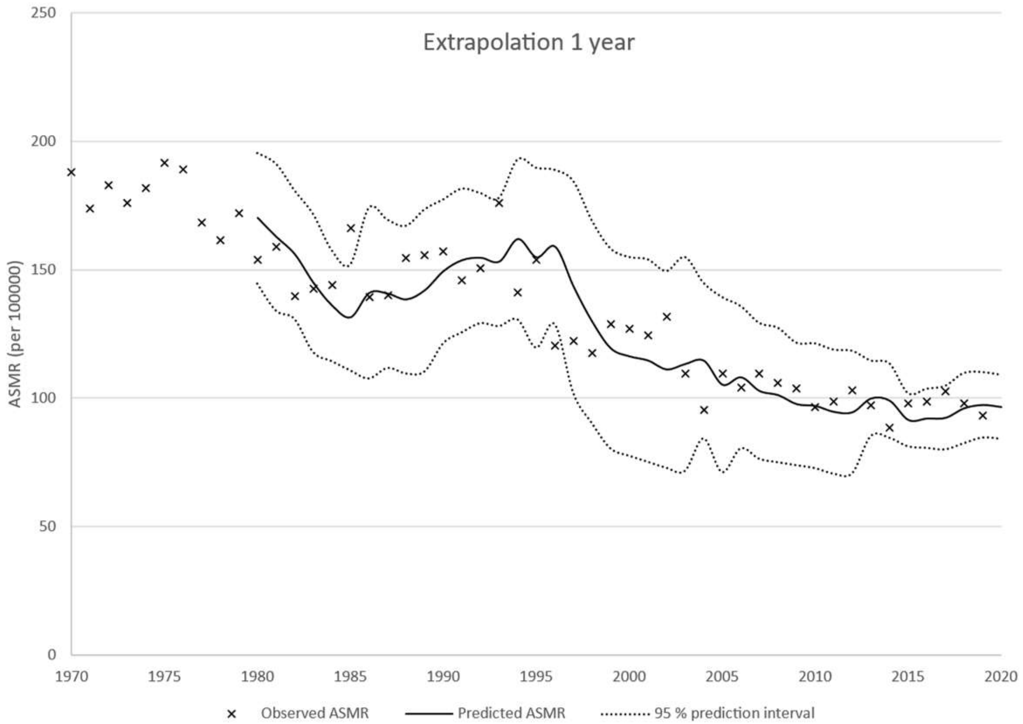

The figures display observed ASMR compared with moving predictions with 95% prediction intervals (PI) based on one-, two- or three-year extrapolations from preceding 10-year linear trends.

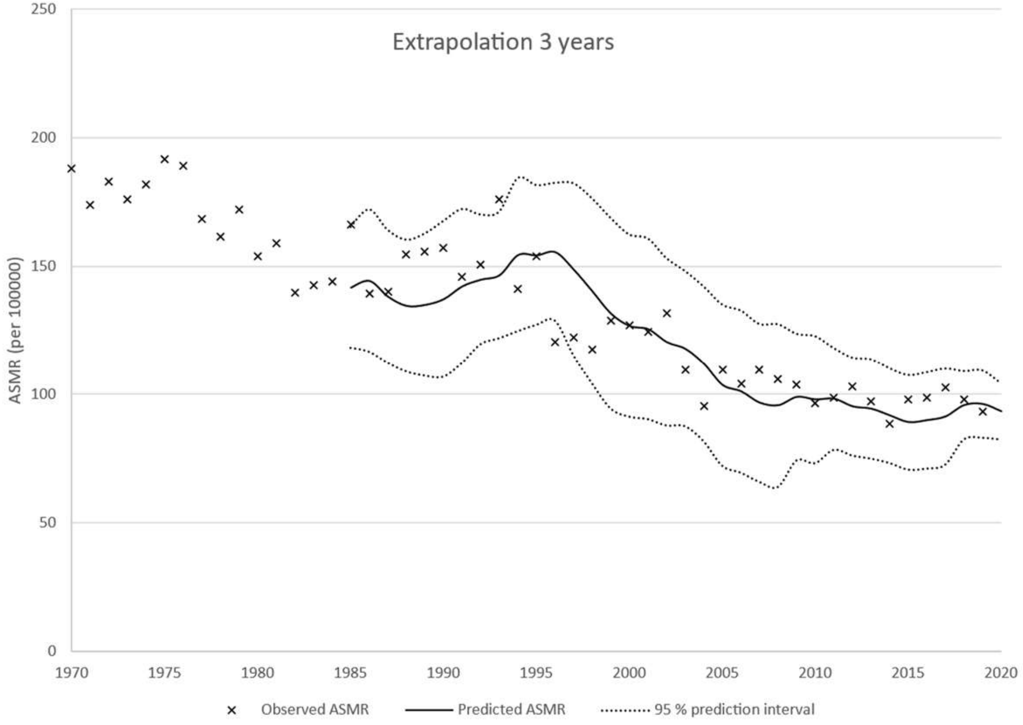

Dementia incl. Alzheimer's

	Extrapolation		
	**1 year**	**2 years**	**3 years**
**Reference years**	-11 to -1	-12 to -2	-13 to-3
Outcome years in study	*2020*	*2021*	*2022*
Reference years in study	*2010-2019*	*2010-2019*	*2010-2019*
**Validation of predictions for 2010 to 2019**			
**MAE%**	3.9%	4.6%	2.8%
**RMSE%**	4.5%	5.5%	4,0%
**Outside 95% prediction interval**	10%	10%	10%

The table shows mean absolute error% (MAE%) and root mean square error% (RMSE%) for age standardised mortality rate (ASMR) predictions for the ten years preceding the COVID-19 pandemic, ie 2010-2019.

Predictions are based on extrapolation from linear 10-year trend until one-, two- or three years prior to each year predicted.

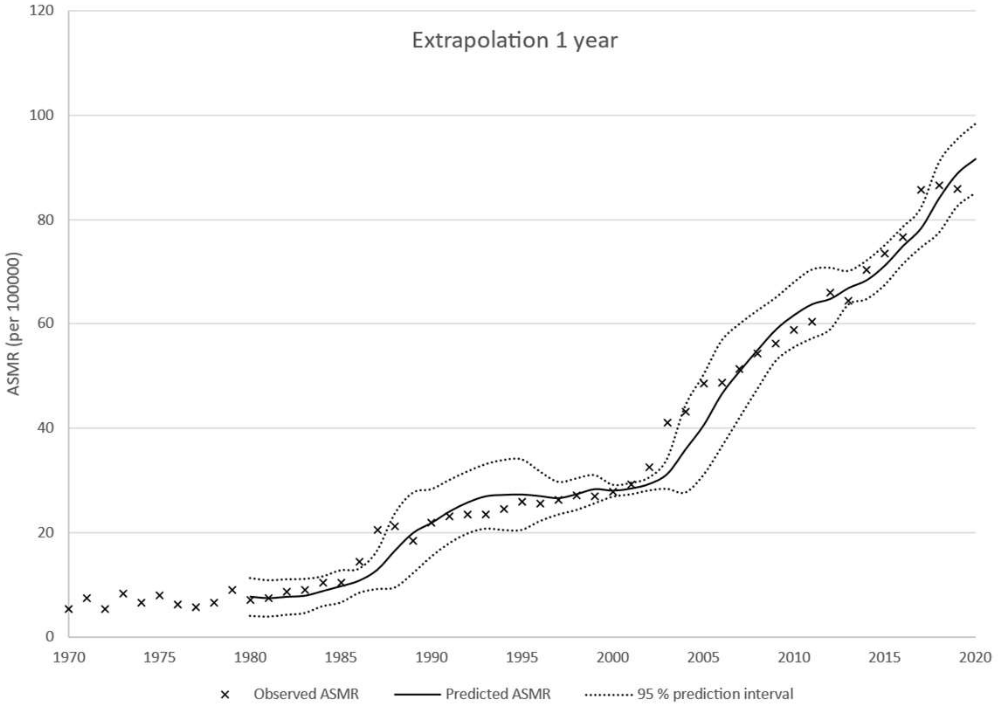

The figures display observed ASMR compared with moving predictions with 95% prediction intervals (PI) based on one-, two- or three-year extrapolations from preceding 10-year linear trends.

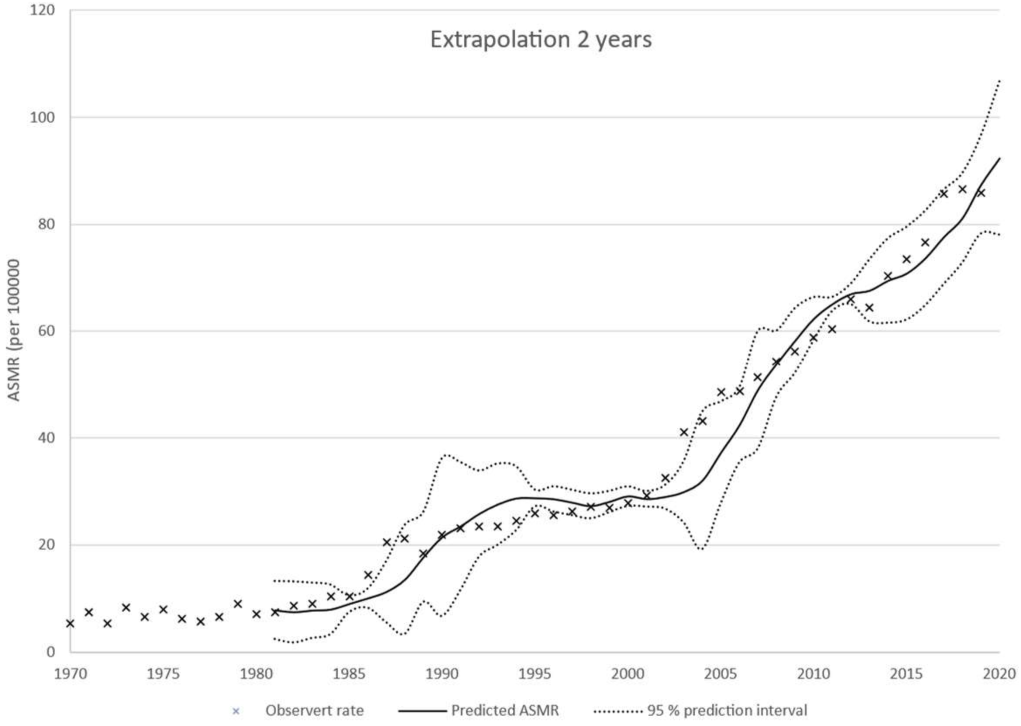

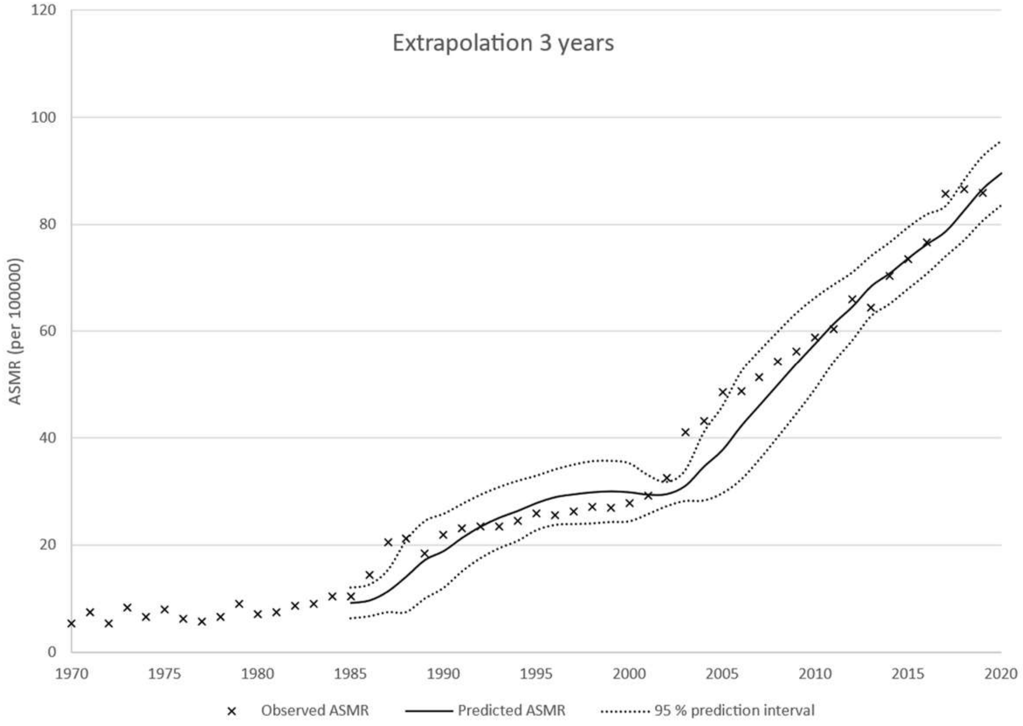

External causes

	Extrapolation		
	**1 year**	**2 years**	**3 years**
**Reference years**	-11 to -1	-12 to -2	-13 to-3
Outcome years in study	*2020*	*2021*	*2022*
Reference years in study	*2010-2019*	*2010-2019*	*2010-2019*
**Validation of predictions for 2010 to 2019**			
**MAE%**	2.8%	3.1%	2.6%
**RMSE%**	3.8%	3.6%	3.4%
**Outside 95% prediction interval**	0%	0%	0%

The table shows mean absolute error% (MAE%) and root mean square error% (RMSE%) for age standardised mortality rate (ASMR) predictions for the ten years preceding the COVID-19 pandemic, ie 2010-2019.

Predictions are based on extrapolation from linear 10-year trend until one-, two- or three years prior to each year predicted.

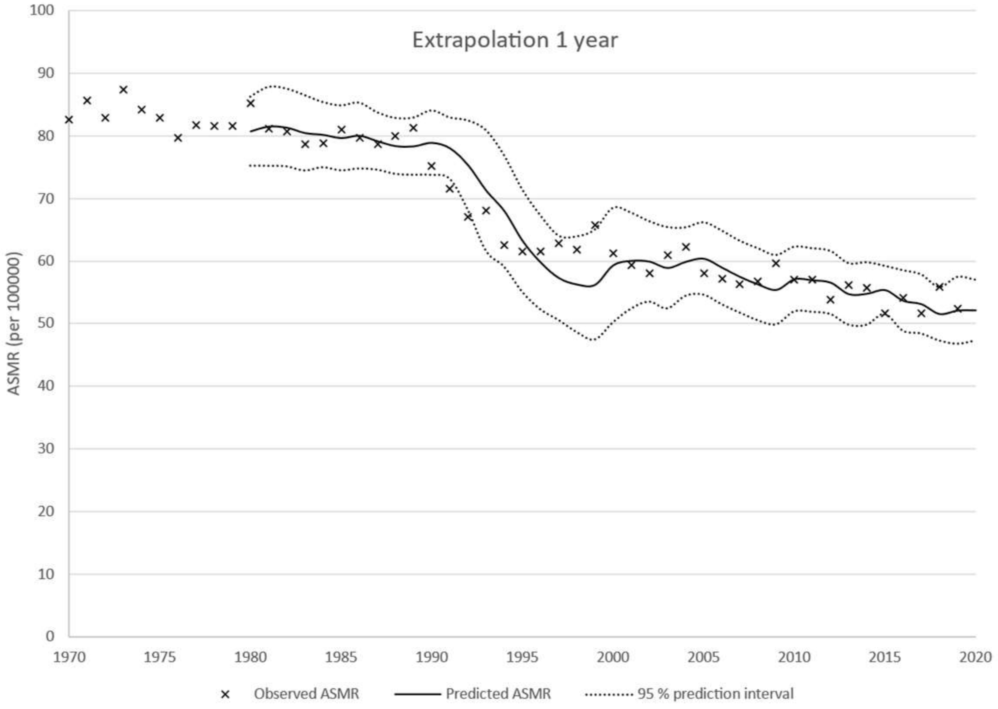

The figures display observed ASMR compared with moving predictions with 95% prediction intervals (PI) based on one-, two- or three-year extrapolations from preceding 10-year linear trends.

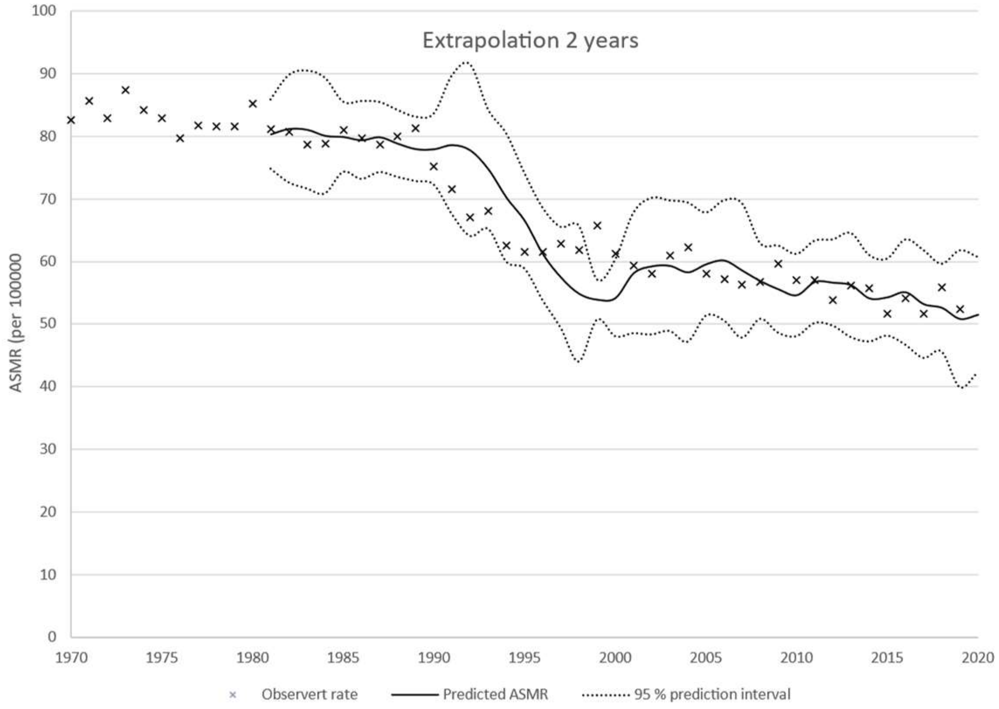

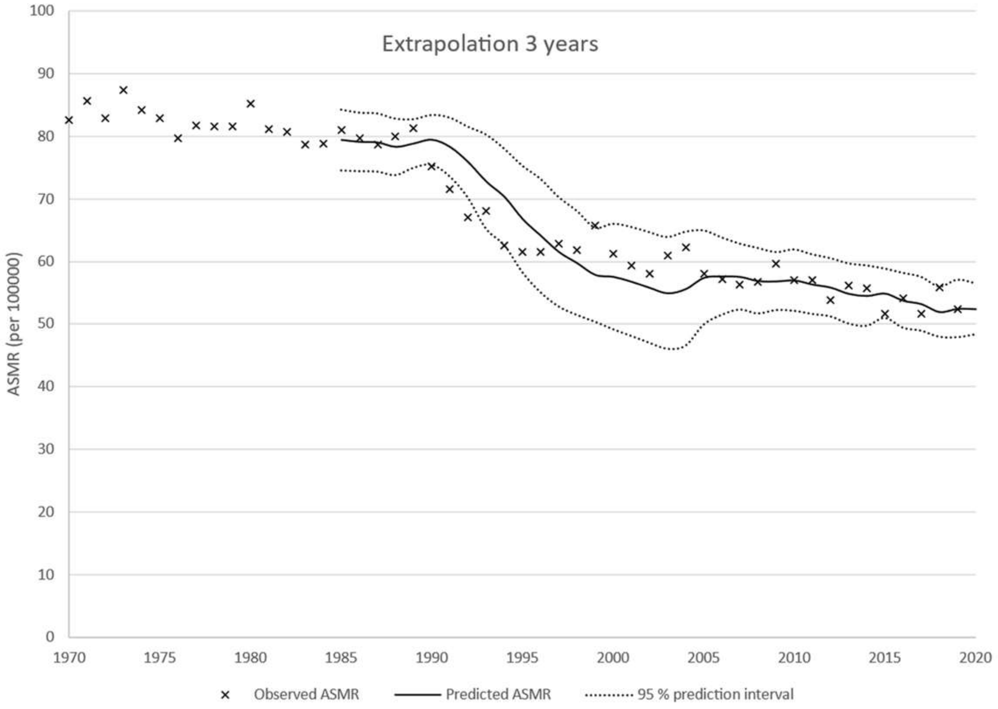

Supplement: Supplementary file 2 — Additional file 2: Supplement 2. Validation of model. Retrospective predictions of age standardised mortality rates (ASMR) with 95% prediction intervals from 1980 to 2019. based on one-, two- or three-year extrapolations from preceding 10-year linear trends. Mean absolute error% (MAE%) and root mean square error% (RMSE%) for 2010-2019 ASMR predictions. [file 12889_2023_17515_MOESM2_ESM.pdf]
